# Improving Adolescent Sexual and Reproductive Health: A Systematic Review of Potential Interventions

**DOI:** 10.1016/j.jadohealth.2016.05.022

**Published:** 2016-10

**Authors:** Rehana A. Salam, Anadil Faqqah, Nida Sajjad, Zohra S. Lassi, Jai K. Das, Miriam Kaufman, Zulfiqar A. Bhutta

**Affiliations:** a Division of Women and Child Health, Aga Khan University, Karachi, Pakistan; b Robinson Research Institute, University of Adelaide, Adelaide, Australia; c Division of Adolescent Medicine, The Hospital for Sick Children and University of Toronto, Toronto, Canada; d Centre for Global Child Health, The Hospital for Sick Children, Toronto, Canada; e Center of Excellence in Women and Child Health, The Aga Khan University, Karachi, Pakistan

**Keywords:** Adolescent sexual health, Reproductive health, Genital mutilation, Sexual health education, Teenage pregnancy, Contraception

## Abstract

Adolescents have special sexual and reproductive health needs (whether or not they are sexually active or married). This review assesses the impact of interventions to improve adolescent sexual and reproductive health (including the interventions to prevent female genital mutilation/cutting [FGM/C]) and to prevent intimate violence. Our review findings suggest that sexual and reproductive health education, counseling, and contraceptive provision are effective in increasing sexual knowledge, contraceptive use, and decreasing adolescent pregnancy. Among interventions to prevent FGM/C, community mobilization and female empowerment strategies have the potential to raise awareness of the adverse health consequences of FGM/C and reduce its prevalence; however, there is a need to conduct methodologically rigorous intervention evaluations. There was limited and inconclusive evidence for the effectiveness of interventions to prevent intimate partner violence. Further studies with rigorous designs, longer term follow-up, and standardized and validated measurement instruments are required to maximize comparability of results. Future efforts should be directed toward scaling-up evidence-based interventions to improve adolescent sexual and reproductive health in low- and middle-income countries, sustain the impacts over time, and ensure equitable outcomes.

A significant number of adolescents around the globe are sexually active, and this proportion increases steadily from mid- to late adolescence [1]. Sexual activity of adolescents varies markedly by gender and region; more girls compared with boys are sexually active in sub-Saharan Africa, Asia, and central Asia while in Latin America and Caribbean, more boys are sexually active than girls [1]. About 3 in 10 unmarried adolescent women in sub-Saharan Africa and nearly one in four in South America have ever had sex [2]. Early sexual debut increases the risk of sexually transmitted infections (STIs), including HIV, and can result in unintended pregnancy and early childbearing. Adolescents have limited and, in some places, no access to sexual and reproductive health education and contraception, making adolescent girls more prone to early and unintended pregnancies [3].

Nearly a quarter of girls aged 15–19 years are married with an estimated 16 million adolescents giving birth each year globally, of whom, 95% are from low- and middle-income countries (LMICs) [4]. Almost half of the women aged 20–24 years in Asia and Africa are married by age 18 years, putting them at a higher risk for early pregnancy, repeated pregnancies, maternal disability, and death [3], [5]. Adolescent birth rate in LMICs is more than double that of high-income countries (HICs) and often within a formal marital relationship, especially in Asia, Middle East, and North African regions [6]. Although rates of births among adolescent girls have declined in all regions since 1990, they are still high in Africa, Asia, Latin America, and Caribbean. Among females aged 15–19 years, pregnancy-related death is the second leading cause of death after self-harm [7]. Younger mothers are at an increased risk of obstetric fistula, anemia, eclampsia, postpartum hemorrhage, and puerperal endometritis [7], [8], [9]. Girls younger than 19 years have a 50% increased risk of stillbirths and neonatal deaths, as well as an increased risk for preterm birth, low birth weight, and asphyxia [8]. In addition to affecting the health of the mother, early marriage and/or childbearing also often prevent girls from attending school and perpetuate the cycle of poverty [9], [10], [11]. In LMICs, adolescent pregnancy is a severe impediment to development and can lead to a number of challenges including abandonment by their partners, school dropout, and lost productivity, which ultimately limits their future social and economic opportunities leading to intergenerational transmission of poverty [12], [13].

Female genital mutilation/cutting (FGM/C) is a hazardous traditional practice on prepubescent girls that involves partial or total removal of the external female genitalia or other injury to the female genital organs for nonmedical reasons [14]. It is practiced in about 28 countries of Africa, and recent figures suggest a prevalence of more than 70% in Burkina Faso, Djibouti, Egypt, Eritrea, Ethiopia, Guinea, Mali, Mauritania, Northern Sudan, Sierra Leone, and Somalia [15], [16]. It is also practiced by immigrant communities in a number of other countries, including Australia, Canada, France, New Zealand, Norway, Sweden, Switzerland, the United Kingdom, and the U.S. [17]. However, there is considerable variation in prevalence between and within countries, reflecting ethnicity and tradition. Girls exposed to FGM/C are at risk of immediate physical consequences, such as severe pain, bleeding, shock, difficulty in passing urine and feces, and infections. Long-term consequences can include chronic pain, sexual/orgasmic dysfunction, infections, and mental trauma [18], [19].

In 2011, the World Health Organization (WHO) issued guidelines on preventing early pregnancy and poor reproductive outcomes in adolescents from LMICs focusing on four major pregnancy prevention outcomes: (1) increasing access to and use of contraception; (2) preventing marriage before 18 years; (3) increasing knowledge and understanding of the importance of early pregnancy prevention; and (4) preventing coerced sex [20].

Adolescents have special sexual and reproductive health needs that remain unmet, mainly due to lack of knowledge, social stigma, laws and policies preventing provision of contraception and abortion to unmarried (or any) adolescents, and judgmental attitudes among service providers [21]. To maintain sexual and reproductive health, adolescents need access to accurate information and to the safe, effective, affordable, and acceptable contraception method of their choice. They must be informed and empowered to protect themselves from STIs. All sexually active adolescents, regardless of marital status, deserve to have their contraceptive needs acknowledged and responded to. This article is part of a series of reviews conducted to evaluate the effectiveness of potential interventions for adolescent health and well-being. A detailed framework, methodology, and other potential interventions have been discussed in separate articles [22], [23], [24], [25], [26], [27], [28]. This article aims to assess the impact of interventions to improve sexual and reproductive health, prevent adolescent pregnancy; FGM/C; and intimate partner violence.

## Methods

We systematically reviewed all published literature up to December 2014 on interventions to improve sexual health in adolescent population focusing on sex education, preventing unintended adolescent pregnancy, intimate partner violence, and FGM/C. We took a systematic approach to consolidate the existing evidence through the following three methodologies in order to include all the recent evidence:

1. Overview of systematic reviews: We conducted an overview of systematic reviews for interventions where recent systematic reviews existed;
2. Updating existing reviews: We updated the existing systematic reviews if the existing review only included evidence prior to 2011; and
3. De novo review: For interventions where no reviews existed, we conducted a de novo review.

For the purpose of this review, the adolescent population was defined as aged 11–19 years; however, since many studies targeted youth (aged 15–24 years) along with adolescents, exceptions were made to include studies targeting adolescents and youth. Studies were excluded if they targeted age groups other than adolescents and youth or did not report segregated data for the age group of interest. The search was conducted till December 2014, and we did not apply any limitations on the start search date or geographical settings and have attempted to carry out subgroup analysis for various interventions and settings, where data permitted.

### Methodology for de novo review

For de novo reviews, our priority was to select existing randomized, quasi-randomized, and before/after studies, in which the intervention was directed toward adolescents and related to sexual and reproductive health outcomes. A separate search strategy was developed for each aspect using appropriate keywords, medical subject heading, and free text terms. The following principal sources of electronic reference libraries were searched to access the available data: The Cochrane Library, Medline, PubMed, Popline, LILACS, CINAHL, EMBASE, World Bank's JOLIS search engine, CAB Abstracts, British Library for Development Studies BLDS at Institute of Development Studies, the WHO regional databases, Google, and Google Scholar. The titles and abstracts of all studies identified were screened independently by two reviewers for relevance and matched. Any disagreements on selection of studies between these two primary abstractors were resolved by the third reviewer. After retrieval of the full texts of all the studies that met the inclusion/exclusion criteria, data from each review or study were abstracted independently and in duplicate into a standardized form. Quality assessment of the included randomized controlled trials (RCTs) was done according to the Cochrane risk of bias assessment tool. We conducted a meta-analysis for individual studies using the software Review Manager, version 5.3 (Cochrane Collaboration, London, United Kingdom). Pooled statistics were reported as the relative risk (RR) for categorical variables and standard mean difference (SMD) for continuous variables between the experimental and control groups with 95% confidence intervals (CIs). A grade of “high,” “moderate,” “low,” and “very low” was used for grading the overall evidence indicating the strength of an effect on specific health outcome according to the Grading of Recommendations Assessment, Development and Evaluation criteria [29].

### Methodology for existing systematic review

We considered all available published systematic reviews on the interventions to improve adolescent sexual health. Our priority was to select existing Cochrane and non-Cochrane systematic reviews of randomized or non-RCTs, which fully or partly addressed the interventions. A broad search strategy was used that included a combination of appropriate keywords, medical subject heading, and free text terms, and search was conducted in The Cochrane Library, Medline, and PubMed. The abstracts (and the full sources where abstracts are not available) were screened by two abstractors to identify systematic reviews adhering to our objectives. Any disagreements on selection of reviews between these two primary abstractors were resolved by the third reviewer. After retrieval of the full texts of all the reviews that met the inclusion/exclusion criteria, data from each review were abstracted independently and in duplicate into a standardized form. Information was extracted on (1) the characteristics of included studies; (2) description of methods, participants, interventions, and outcomes; (3) measurement of treatment effects; (4) methodological issues; and (5) risk of bias tool. We extracted pooled effect size for the outcomes of interest with 95% CIs. We assessed and reported the quality of included reviews using the 11-point assessment of the methodological quality of systematic reviews (AMSTAR) criteria [30].

### Methodology for updated review

We updated the existing systematic reviews only if the most recent review on a specific intervention was conducted before December 2011. For updating the existing reviews, we adopted the same methodology and search strategy mentioned in the existing review to update the search and find all the relevant studies after the last search date of the existing review. After retrieval of the full texts of all the articles that met the inclusion/exclusion criteria, data from each study were abstracted independently and in duplicate into a standardized form. Information was extracted on study design, geographical setting, intervention type and description, mode of delivery, and outcomes assessed. We then updated the estimates of reported outcomes by pooling the evidence from the new studies identified in the updated search and reported new effect size for the outcomes of interest with 95% CIs. We then assessed and reported the quality of included reviews using the 11-point AMSTAR criteria [30].

## Results

**Figure 1:**
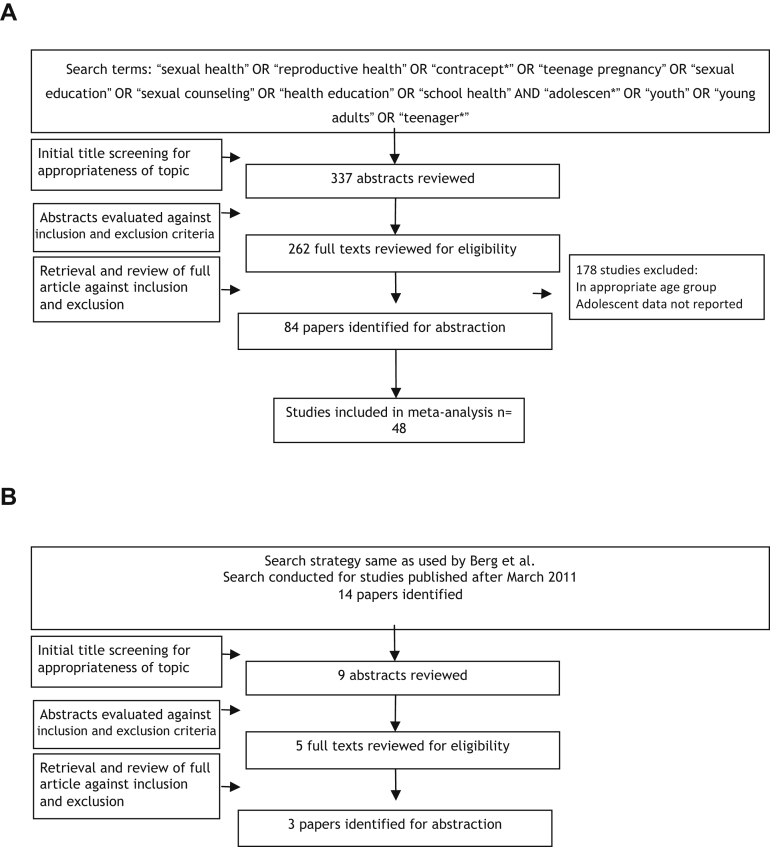
(A) Search flow for interventions to improve sexual and reproductive health and prevent adolescent pregnancy (de novo review). (B) Search flow for interventions to prevent FGM/C (update).

We found existing systematic reviews on interventions for improving adolescent sexual and reproductive health; however, they were limited in their scope to a particular strategy such as school-based interventions [31], [32], peer-led interventions [33], mass media [34], [35], and youth centers [36]; geographic settings [37], [38]; or limited to trial data only [13], [39]. Hence, we conducted a de novo review for the effectiveness of sexual and reproductive health education and contraceptive availability. We found a recent existing Cochrane review by Fellmeth et al. [40] on interventions to prevent intimate partner violence and reported the relevant findings. For interventions to prevent FGM/C, we updated the review by Berg and Denison [14] and also broadened its scope to include studies outside of Africa. Figure 1 depicts the search flow diagrams while Table 1 describes in detail the characteristics of the included studies for the de novo review.

### Sexual and reproductive health interventions to prevent adolescent pregnancy

Studies were included if any form of sexual and reproductive health education, counseling, and access to contraception was delivered to adolescents compared to no intervention or general health education. We identified 1,123 titles from the search conducted in all databases. After screening the titles and abstracts, 84 studies were identified that met the inclusion criteria [41], [42], [43], [44], [45], [46], [47], [48], [49], [50], [51], [52], [53], [54], [55], [56], [57], [58], [59], [60], [61], [62], [63], [64], [65], [66], [67], [68], [69], [70], [71], [72], [73], [74], [75], [76], [77], [78], [79], [80], [81], [82], [83] [84], [85], [86], [87], [88], [89], [90], [91], [92], [93], [94], [95], [96], [97], [98], [99], [100], [101], [102], [103], [104], [105], [106], [107], [108], [109] [110], [111], [112], [113], [114], [115], [116], [117], [118], [119], [120], [121], [122], [123], [124]; 51 studies were RCTs while 29 were quasi-experimental design and four were pre–post studies. Fifty four of 84 studies focused on adolescent age group alone (11–19 years) while the rest had overlapping age groups. Meta-analysis could be conducted for 48 studies as other studies did not report data that could be pooled. Most of these studies were conducted in HICs in North America and Europe except 10 studies that were conducted in LMICs including Zambia, Zimbabwe, Cameroon, Tanzania, Gambia, Kenya, China, and Peru. Interventions mainly included (1) education and counseling through peer groups, parent education, community members, telephone calls, Web-based content, and home visitation; (2) youth-friendly health services; (3) improving access to contraceptives through pharmacy, clinic, and advance provision of contraceptives; (4) condom distribution; (5) abstinence-focused education; (6) emergency contraceptive promotion; (7) skills development; and (8) multicomponent interventions.

**Figure 2:**
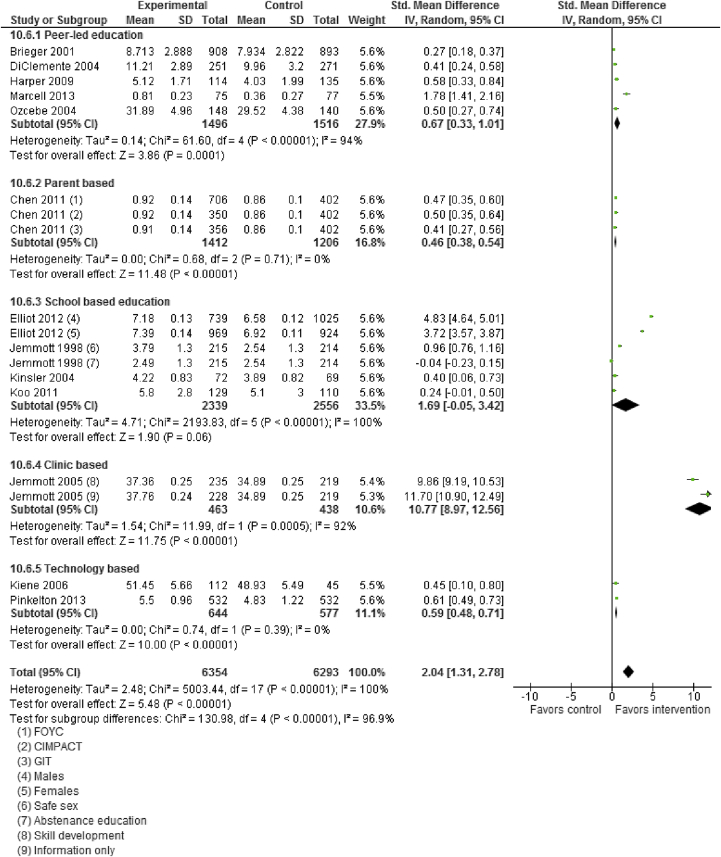
Impact of sexual health education/counseling on mean knowledge score. CI = confidence interval; FOYC = Focus on Youth in the Caribbean; GIT = Goal for IT; IV = inverse variance; SD = standard deviation.

**Figure 3:**
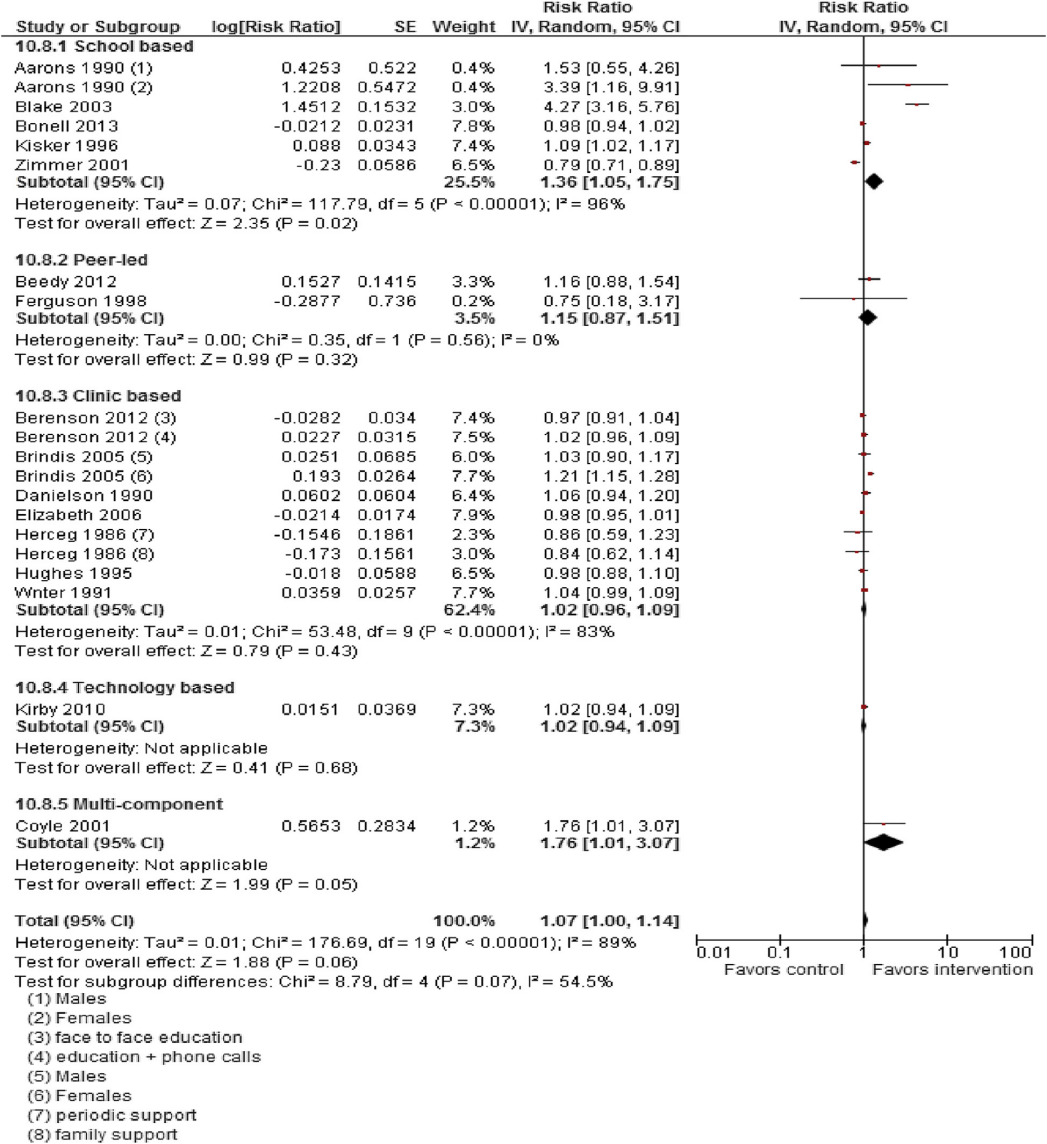
Impact of sexual health education/counseling on use of any contraception. CI = confidence interval; IV = inverse variance; SE = standard error.

Moderate quality data suggest that sexual and reproductive health education, counseling, and contraceptive availability increased “mean knowledge score about sexual health and contraception” (SMD: 2.04; 95% CI: 1.31–2.78), “mean condom use self-efficacy score” (SMD: .76; 95% CI: .22–1.30), use of any contraception (RR: 1.07; 95% CI: 1.00–1.14), and condom use (RR: 1.11; 95% CI: 1.04–1.20; Figures 2 and 3). Sexual health education did not significantly impact risk of having sex (RR: 1.00; 95% CI: .93–1.07) or STIs (RR: 1.08; 95% CI: .79–1.46). Pooled analysis from moderate quality evidence showed a 15% decrease (RR: .85; 95% CI: .74–.98) in incidence of adolescent pregnancies and a 37% decrease (RR: .63; 95% CI: .49–.82) in the rate of repeat adolescent pregnancies.

Subgroup analysis according to the type of interventions suggests that peer-led counseling significantly improved mean knowledge score however did not significantly impact use of contraception. Peer-led counseling comprised peer educators providing information and counseling through one-on-one sessions, group talks and presentations, and distribution of print materials. Number of group sessions varied from study to study, ranging from three sessions to nine sessions. The intervention mainly emphasized male and female anatomy–physiology of the reproductive system, preventive precautions against sexually transmitted diseases and HIV/AIDS, family planning methods, communication skills, ethnic and gender pride, condom use skills, and healthy relationships. Parent-directed interventions were also effective in improving sexual knowledge, and the interventions included a 20-minute video filmed, which addressed decision-making regarding future planning for the parent and child, parent–child communication about sexual decision-making, followed by role-playing and a condom demonstration. The video was followed by a structured discussion among the participants. Clinic-based interventions comprising counseling, skills building, and case management services improved mean knowledge. These findings are limited to a single study only. Clinic-based face-to-face behavioral counseling and education followed by monthly phone calls for 6 months and reproductive health intervention combining a highly explicit half-hour slide-tape program with a personal health consultation did not have any impact on contraceptive use. Technology-based interventions including custom-computerized intervention in which content and delivery were based on the Information–Motivation–Behavioral Skills model of health behavior change and teen-led, media literacy curriculum focused on sexual portrayals in the media were effective in improving sexual knowledge but did not have any impact on contraceptive use. School-based interventions including combined sex education with youth-friendly sexual health services, curriculum modules implemented by adult facilitators or peer cofacilitators (including abstinence education, delaying sexual intercourse or reducing its frequency, safer sex intervention, condom use), and HIV prevention education were effective in improving contraceptive use but did not impact mean knowledge scores. Subgroup analysis for HICs and LMICs could not be conducted due to limited number of studies in LMIC settings (Table 2). Data quality was rated to be “moderate” since the study designs were not robust (included RCTs, quasi and pre–post studies), substantial statistical heterogeneity, and limited generalizability.

### Female genital mutilation

**Figure 4:**
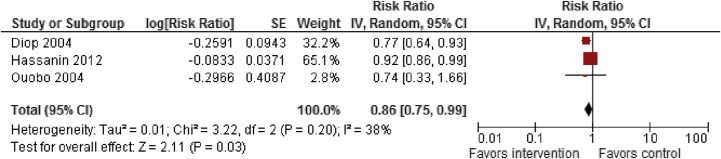
Impact of interventions to prevent FGM on FGM prevalence. CI = confidence interval; FGM = female genital mutilation; IV = inverse variance; SE = standard error.

**Figure 5:**
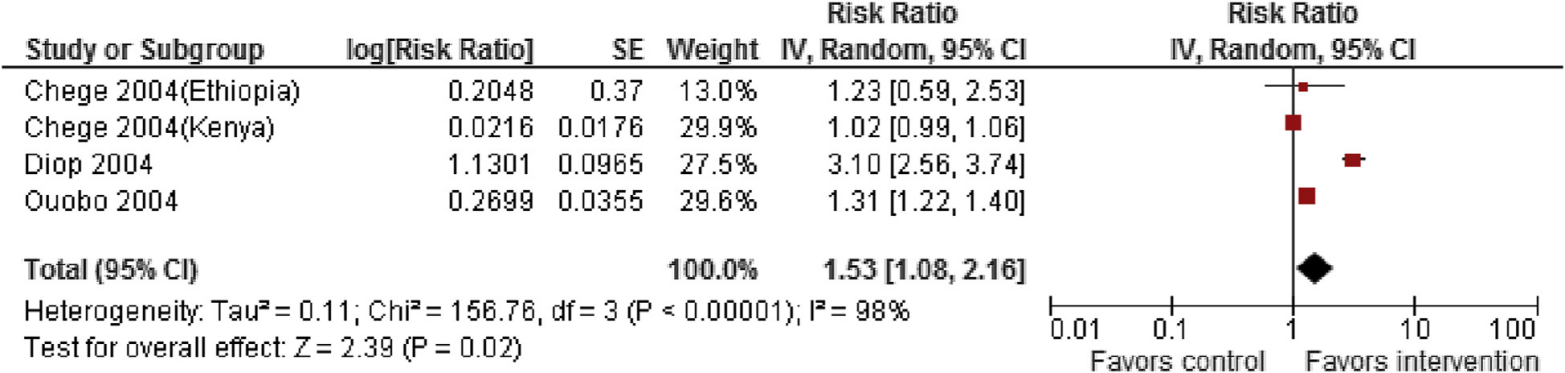
Impact of interventions to prevent FGM on knowledge of harmful consequences. CI = confidence interval; FGM = female genital mutilation; IV = inverse variance; SE = standard error.

A search was conducted for literature published after March 2011 following the same methodology as Berg et al. A total of 11 studies [125], [126], [127], [128], [129], [130], [131], [132], [133], [134], [135] (eight included in existing review + three new studies) were included, mostly from Africa. All studies were pre–post studies. For female genital mutilation prevention, studies focused on interventions, including: (1) legislation against FGM/C; (2) education about health risks associated with FGM/C; (3) training health workers as change agents; (4) training and converting circumcisers; (5) alternative rites; (6) positive deviance; and (7) comprehensive social development including outreach and advocacy. Findings from low-quality evidence suggest that interventions to prevent FGM/C did not have any significant impact on belief that FGM/C compromises human rights of women, though there was significant statistical heterogeneity in the two included studies (RR: 1.30; 95% CI: .47–3.64). However, these interventions significantly reduced the prevalence of FGM/C (RR: .86; 95% CI: .75–.99) and improved knowledge of harmful consequences of FGM/C (RR: 1.53; 95% CI: 1.08–2.16; Figures 4 and 5), though there was significant heterogeneity in the interventions (Table 3). Subgroup analysis suggests that these interventions significantly improved knowledge of harmful consequences in both men and women. These studies suggest that the factors related to the continuance and discontinuance of FGM/C varied across contexts, but the main factors that supported FGM/C were tradition, religion, and reduction of women's sexual desire.

### Intimate partner violence

We report the findings from a Cochrane review by Fellmeth et al. [40] focusing on educational and skills-based interventions targeted at young people aged 12–25 years for preventing intimate partner violence with an AMSTAR rating of 11 . A total of 38 studies were included, 33 of which were included in the meta-analysis. All the included studies were conducted in HICs. There was an increase in knowledge related to relationship violence in favor of the intervention (SMD: .44; 95% CI: .28–.60). However, moderate-quality evidence suggests no significant impact of such interventions on episodes of relationship violence (RR: .77; 95% CI: .53–1.13), behavior scores related to relationship violence (SMD: −.07; 95% CI: −.31 to .16), and a skills score related to relationship violence (to communicate effectively; SMD: .03; 95% CI: −.11 to .17). Subgroup analyses showed no statistically significant differences by intervention setting or type of participants.

## Discussion

Our review suggests that sexual and reproductive health education, counseling, and contraceptive availability are effective in increasing adolescent knowledge related to sexual health, contraceptive use, and decreasing adolescent pregnancy. We could not conduct subgroup analysis for the effectiveness of these interventions in HICs and LMICs since there were limited studies from LMIC settings. Among interventions to prevent FGM/C, community mobilization and female empowerment have the potential to raise awareness of the adverse health consequences of FGM/C and decrease its prevalence; however, there is a need to conduct methodologically rigorous intervention evaluations. Overall, there was limited and inconclusive evidence for the effectiveness of interventions to prevent intimate partner violence.

Our findings are in concordance with existing reviews evaluating the effectiveness of various interventions for improving adolescent sexual and reproductive health and also collate various interventions under a broader umbrella to evaluate the combined effectiveness of these interventions. An existing Cochrane review on primary prevention interventions (school based, community or home based, clinic based, and faith based) on unintended pregnancies among adolescents also suggests that combination of educational and contraceptive interventions can lower the rate of unintended pregnancy among adolescents with nonconclusive evidence on secondary outcomes, including initiation of sexual intercourse, use of birth control methods, abortion, childbirth, and STIs [13]. Group-based comprehensive risk reduction has been reported as an effective strategy to reduce adolescent pregnancy, HIV, and STIs while effectiveness of group-based abstinence education was inconclusive [136]. Another review on adolescent fertility in LMICs suggests improved knowledge-based indicators in the intervention groups of almost all interventions evaluated; however, it is not clear that such interventions necessarily lead to short- or long-term behavior change [137], [138].

The United Nations Fund for Population Activities (UNFPA) and United Nations International Children's Emergency Fund (UNICEF) joint program, developed in 2007 to protect girls and women by accelerating abandonment of FGM/C and providing care for its consequences, has accelerated existing changes toward FGM/C abandonment by legal frameworks, coordination mechanisms, and access to services at both community and national level. But, further efforts are needed, especially at the national and community levels, to bring changes in behaviors and practices [139]. A recent report by WHO on preventing intimate partner and sexual violence suggests that evidence is still in its infancy and much remains to be accomplished [140].

This existing evidence on adolescent sexual reproductive health has several limitations. Most trials failed to utilize allocation concealment, blinding, and randomization to optimize their outcomes. Hence, most of the outcomes were rated as low or moderate in methodological quality. There was a lack of rigorous study design for the interventions to prevent FGM/C with most studies utilizing before and after designs without comparable controls, although individual or cluster RCTs to address FGM/C would pose huge ethical challenges. Nevertheless, many of the trials focused on nonstandardized and self-reported outcomes with short follow-up periods that might have been insufficient to detect any meaningful behavioral changes to establish or to wash out the effect of intervention. Most studies on intimate partner violence analyzed outcomes such as attitude and knowledge rather than episodes of violence and behavioral change. Furthermore, we found a dearth of evidence on interventions for improving sexual health of adolescents living in LMICs where the majority of the adolescent population of the world resides. This might lead to limited external validity for many of these interventions. Most of the studies did not report data segregated by gender which is essential since males and females might respond differently to behavioral interventions. The wide variability in study constructs, nonuniformity in subgroup population, lack of subgroup analysis of gender, socioeconomic status, and nonstandardized outcomes all preclude the external validity and effectiveness of the present interventions in LMICs.

Our review suggests that a range of comprehensive interventions targeting sexual health education, counseling, consistent birth control methods promotion, and provision have the potential to prevent and control the adverse outcomes related to risky sexual behavior. However, much more is needed to increase awareness and prevent FGM and intimate partner violence.

## Figures and Tables

**Table 1:** Characteristics of included studies for sexual health education

Author, year	Design	Country	Setting	Intervention	Target population	Outcomes
Aarons et al., 2000 [41]	RCT	U.S.A.	School based	Reproductive health classes, the Postponing Sexual Involvement curriculum, health risk screening, and “booster” educational activities during the following (eighth grade) school year.	Eighth-grade students	Use of contraception
Agha and Van Rossem, 2004 [42]	Quasi	Zambia	Community based	Peer-led education projects focusing on promoting abstinence and condom use among male and female school children. Peer educators consisted of people aged 18–22 years who were trained by a professional peer education trainer to convey their messages through a mixture of techniques.	Males and females aged 14–23 years	Abstinence, multiple sexual partners, condom use
Baptiste et al., 2006 [43]	RCT	South Africa and Trinidad and Tobago	Community based	Community participatory research framework to adapt and deliver family-based prevention.	Youth	Communication, HIV knowledge and prevalence
Barnet et al., 2007 [44]	RCT	U.S.A.	Home based	Home visiting or routine care. They delivered a parenting curriculum, encouraged contraceptive use, connected the teen with primary care, and promoted school continuation.	Pregnant females aged 12–18 years	Birth control after index child, repeat pregnancy
Berenson and Rahman, 2012 [45]	RCT	U.S.A.	Clinic	Face-to-face behavioral counseling and education at their baseline clinic visit or this same intervention followed by monthly phone calls for 6 months or standard care.	Females aged 16–24 years	Consistent contraceptive use, hormonal contraceptive use, condom use, dual usage, no contraception
Morrison-Beedy et al., 2012 [94]	RCT	U.S.A.	Community based	Peer education groups. Theory-based sexual risk reduction intervention or a structurally equivalent health promotion control group.	Females aged 15–19 years	Multiple sexual partners, intercourse
Black et al., 2006 [46]	RCT	U.S.A.	Home based	Home visiting or routine care. Home-based intervention curriculum was based on social cognitive theory and focused on interpersonal negotiation skills, adolescent development, and parenting.	Postpartum females 13.5–17.9 years	Repeat pregnancy
Blake et al., 2003 [47]	Quasi	U.S.A.	School based	Condom availability in high school	High-school adolescents	Condom use
Bonell et al., 2013 [48]	RCT	England	Community based	“Teens and toddlers” intervention: 18–20 weekly sessions in preschool nurseries	Teens	Contraception use
Brieger et al., 2001 [49]	Quasi	West Africa: Nigeria and Gambia	Community based	Peer education groups. Peer educators provided information and counseling through one-on-one sessions, group talks and presentations, and distribution of print materials.	Male and female adolescents	Knowledge
Bull et al., 2012 [50]	RCT	U.S.A.	Community social media	Exposure to Just/Us, a Facebook page developed with youth input, or to control content on 18–24 News, a Facebook page with current events for 2 months.	Youth	Condom use
Chen et al., 2010 [51]	RCT	U.S.A.	Community and school based	“Focus on Youth in the Caribbean” (FOYC) is based on the Protection Motivation Theory (PMT), consists of 10 primary sessions (delivered weekly after a baseline survey) and two annual boosters (delivered after 12 and 24 months postintervention assessment). The sessions are designed to augment decision-making skills, including the development of a lifelong perspective in decision-making, communication and listening skills, and protective knowledge and skills regarding safer sexual behavior. GFI and CImPACT are parenting interventions. GFI includes a 20-minute video filmed in the USA, which addresses decision-making regarding future planning for the parent and child; the video is followed by a structured discussion among the participants. CImPACT includes a 20-minute video filmed in the Bahamas addressing parent–child communication about sexual decision-making, followed by role-playing and a condom demonstration.	Males and females aged 10–12 years	Knowledge, condom use
Clark et al., 2005 [52]	RCT	U.S.A.	School based	The AIM is a 10-session curriculum based on the theory of possible selves. Class exercises encourage students to articulate a possible future self-identity and to develop self-promotion skills.	Seventh-grade African-Americans	Abstinence, sex initiation
Cornelius et al., 2013 [53]	Pre–post	U.S.A.	Community social media	Becoming a Responsible Teen (BART) is a community-based HIV prevention curriculum, which consists of interactive group discussions and role plays that allow participants to practice behavioral skills for safer sex. BART was followed by the delivery of daily multimedia messages for 3 months	African-American adolescents	Knowledge, condom use, HIV prevention scores
Coyle et al., 2001 [54]	RCT	U.S.A.	School based	Safer Choices, a theory-based, multicomponent educational program designed to reduce sexual risk behaviors and increase protective behaviors in preventing HIV, other STDs, and pregnancy among high-school students.	Ninth-grade students	Condom use
Coyle et al., 2004 [56]	RCT	U.S.A.	School based	Draw the Line/Respect the Line, a theoretically based curriculum designed to reduce sexual risk behaviors among middle-school adolescents.	Sixth graders	Sex initiation, knowledge
Coyle et al., 2013 [55]	RCT	U.S.A.	School based	(1) HIV/STI/pregnancy prevention curriculum only; (2) service learning only; (3) HIV/STI/pregnancy prevention curriculum plus service learning; or (4) an attention control curriculum	High-school adolescents	Mean sexual intercourse, mean use of condom
Daniel 2008 [124]	Quasi	India	Community based	Orientation and training of reproductive health teams of community leaders and influential residents, and through group meetings with young couples' parents and in-laws; messages were disseminated through street theater performances and wall paintings, and formal and informal rural health service providers were trained on reproductive health issues and contraception.	Young couples aged <25 years	Contraceptive use, knowledge and attitude
Danielson et al., 1990 [57]	RCT	U.S.A.	Clinic	A reproductive health intervention combining a highly explicit half-hour slide-tape program with a personal health consultation was provided.	Males aged 15–18 years	Consistent contraceptive use, hormonal contraceptive use, condom use, dual usage, no contraception
DiClemente et al., 2004 [58]	RCT	U.S.A.	Community based	Peer education groups. All participants received four 4-hour group sessions. The intervention emphasized ethnic and gender pride, HIV knowledge, communication, condom use skills, and healthy relationships. The comparison condition emphasized exercise and nutrition.	Females aged 14–18 years	Condom use
DiClemente et al., 2009 [59]	RCT	U.S.A.	Clinic	Intervention participants received two 4-hour group sessions and four telephone contacts over a 12-month period, targeting personal, relational, sociocultural, and structural factors associated with adolescents' STD/HIV risk and were given vouchers facilitating male partners' STD testing/treatment.	Females aged 15–21 year	Condom use
Dilorio et al., 2007 [60]	RCT	U.S.A.	Community based	HIV education, communication skills, take-home activities for fathers and adolescents.	Males aged 11–14 years	Condom use
Downs et al., 2004 [61]	RCT	U.S.A.	Community based	Video-based sessions versus book-based information	Urban adolescent girls	Abstinence, condom use, STI
Elliott et al., 2012 [62]	Quasi	Scotland	School based	Healthy Respect 2 (HR2) combined sex education with youth-friendly sexual health services, media campaigns and branding, and encouraged joint working between health services, local government and the voluntary sector.	15- to 16-year-old adolescents	Knowledge, condom acceptability, having sex, condom use
Erulkar et al., 2004 [121]	Quasi	Kenya	Community based	The Nyeri Youth Health Project, a community-based project for young people. Adult counselors worked in their own communities to educate both adolescents and parents on reproductive health and to encourage dialogue between them. The counselors were trained for 1 month and used a life skills curriculum entitled “Life Planning Skills for Adolescents in Kenya,” which includes sessions on community, family and individual values, adolescent development, sexuality, gender roles, relationships, pregnancy, STIs, HIV/AIDS, harmful traditional practices, substance abuse, planning for the future, children's rights, and advocacy.	10–24 years of age	Sexual initiation, secondary abstinence, condom use, sex partners, communication with parents
Ferguson, 1998 [63]	RCT	U.S.A.	Community based	Peer education groups. Peer counseling in a culturally specific adolescent pregnancy prevention program.	Females aged 12–16 years	Condom use
Forehand et al., 2007 [64]	RCT	U.S.A.	Community	Enhanced communication intervention (five sessions), single-session communication intervention(one session)	African-American parent–preadolescent dyads (child, aged 9–12 years)	Mean change in knowledge
Garcia et al., 2012 [65]	RCT	Peru	Community based	The intervention comprised four modalities: strengthened STI syndromic management by pharmacy workers and clinicians; mobile-team outreach for STI screening and pathogen-specific treatment; periodic presumptive treatment of FSWs for trichomoniasis; and condom promotion.	Urban young men (aged 18–29 years) and female sex workers	Sexual intercourse, STI prevalence
Gold et al., 2004 [66]	RCT	U.S.A.	Clinic based	The intervention group received information about EC and was told how to access EC and in addition received one complete course of EC for future use. Participants in the AEC group were informed that they could obtain up to two additional courses of advance EC during the 6 months of the study and were told that they could obtain these subsequent courses from the study office whenever they requested them. Participants in the control group received written and verbal information about EC and were told how to access EC on request from the adolescent clinic.	Urban adolescent females aged 15–20 years	EC use
Hall et al., 2012 [67]	RCT	U.S.A.	Community text messaging	The intervention group received 180 daily text messages, including 47 individual messages (which were repeated up to four times over the study).	Young women aged 13–25 years	OC knowledge
Harper et al., 2009 [68]	Quasi	U.S.A.	Community based	Peer education groups. Nine-session SHERO's (a female-gendered version of the word hero) intervention or a single-session information-only HIV prevention intervention.	Females aged 12–21 years	Intercourse, knowledge
Herceg-Baron et al., 1986 [69]	Quasi	U.S.A.	Clinic	Increase family + teenager or teenager + clinic staff contact	Adolescent aged 12–17 years	Contraceptive use, unintended pregnancy
Howard and McCabe, 1990 [70]	Quasi	U.S.A.	Community based	Peer education. The program is led by older teenagers and focuses on helping students resist peer and social pressures to initiate sexual activity.	Males and females aged 14–16 years	Intercourse
Hughes et al., 1995 [71]	RCT	U.S.A.	Clinic	Increase in family planning clinic for education counseling and access compared with no clinic.	Females aged 14–18 years	Unintended pregnancy, intercourse
Jemmott III et al., 1998 [72]	RCT	U.S.A.	School based	HIV and abstinence education: intervention involved eight 1-hour modules implemented by adult facilitators or peer cofacilitators. Abstinence education enforced delaying sexual intercourse or reducing its frequency; safer sex intervention stressed condom use; control intervention concerned health issues unrelated to sexual behaviors.	African-American adolescents	Having sex, condom use, knowledge, self-efficacy
Jemmott et al., 2005 [73]	RCT	U.S.A.	Clinic	Counseling, skills building, and case management services	Females aged 12–19 years	Knowledge, number of sex partners, unprotected intercourse
Jemmott et al., 2010 [74]	RCT	South Africa	School based	Two 6-session interventions based on behavior-change theories and qualitative research. The HIV/STD risk-reduction intervention targeted sexual risk behaviors; the attention-matched health promotion control intervention targeted health issues unrelated to sexual behavior.	Sixth-grade students	Having sex, multiple sex partners, condom use
Mason-Jones et al., 2011 [89]	Quasi	U.S.A.	Community based	Peer-led education sessions. Peer educators were recruited and trained to provide information and support to their fellow students.	Males and females aged 15–16 years	Abstinence, condom use
Key et al., 2008 [75]	Quasi	U.S.A.	School based	School-based social work services coordinated with comprehensive health care.	Teen mothers	Contraception, pregnancy
Kiene and Barta, 2006 [76]	RCT	U.S.A.	Community computer based	Custom computerized intervention. Content and delivery were based on the Information–Motivation–Behavioral Skills model of health behavior change and used Motivational Interviewing techniques.	College students	Knowledge, condom availability and use, sexual risk behavior
Kim et al., 2001 [123]	Quasi	Zimbabwe	Community-based mass media	Multimedia campaign promoted sexual responsibility among young people in Zimbabwe, while strengthening their access to reproductive health services by training providers.	10–24 years of age	Contraceptive knowledge, contraceptive use
Kinsler et al., 2004 [77]	Quasi	U.S.A.	School based	HIV prevention education: Project Light uses a cognitive-behavioral approach to motivate the program participants to change their risk behaviors and adopt safer behaviors.	Primary and secondary school students	Knowledge, efficacy, communication
Kirby et al., 2004 [79]	RCT	U.S.A.	School based	Safer Choices was designed to reduce unprotected sex by delaying initiation of sex, reducing its frequency, or increasing condom use. Its five components included: school organization, an intensive curriculum with staff development, peer resources and school environment, parent education, and school-community linkages.	Ninth-grade adolescents	Sexual risk-taking
Kirby et al., 2010 [78]	RCT	U.S.A.	Clinic- and telephone-based follow-ups	Regular clinic services or regular clinic services plus nine follow-up phone calls over 12 months to improve sexual risk behaviors.	Females aged 14–18 years	Use of contraception
Kisker et al., 1996 [80]	Quasi	U.S.A.	School based	School-Based Adolescent Health Care Program, which provided comprehensive health-related services.	Youth	Health care access, knowledge, sexual risk behaviors
Klepp et al., 1997 [81]	RCT	Tanzania	School based	Sexual and HIV risk reduction and awareness	Sixth graders	Knowledge, sexual initiation
Kogan et al., 2012 [82]	RCT	U.S.A.	Community based	The Strong African American Families–Teen (SAAF–T) program, a family-centered preventive intervention that included an optional condom skills unit.	16-year-old African-American and their caregivers	Unprotected intercourse and condom use efficacy
Koo et al., 2011 [83]	RCT	U.S.A.	School based	10–13 classroom sessions related to delaying sexual initiation	Fifth-grade students	Knowledge, communication, having sex, abstinence
Larkey et al., 2010 [84]	RCT	Tanzania	Community and school based	Provision of youth-friendly health services, as part of a package of interventions including reproductive health education in primary school; the provision of youth-friendly sexual and reproductive health services; community-based condom promotion and distribution; and community-wide activities.	Adolescents 15 years onward and community people	Prevalence of STI, clinic visit and condom distribution
Lewis et al., 2010 [85]	Quasi	Australia	Clinic	Contraceptive education and use of Implanon or depot medroxyprogesterone acetate (DMPA) or nothing	Postpartum females (12–18 years old)	Unintended pregnancy
Lim et al., 2012 [86]	Quasi	Australia, New Zealand	Community text messaging	The 12-month intervention included SMS (catchy sexually transmissible infections prevention slogans) and e-mails.	Youth aged 16–29 years	Having sex, condom use, STI test
Lou et al., 2004 [122]	Quasi	China	Community based	The intervention intended to build awareness and offer counseling and services related to sexuality and reproduction among unmarried youths, in addition to the routine program activities, which were exclusively provided in the control site.	15–24 years of age	Contraceptive use, condom use
Marcell et al., 2013 [87]	Quasi	U.S.A.	Community based	Peer education. Participants received three 1-hour curriculum sessions on consecutive days.	Males aged 16–24 years	Multiple sexual partners, intercourse, condom use, knowledge
Markham et al., 2012 [88]	RCT	U.S.A.	School based	Group and individualized computer-based activities addressing psychosocial variables. The risk avoidance (RA) program met federal abstinence education guidelines; the risk reduction (RR) program emphasized abstinence and included computer-based condom skills training.	African-American and Hispanic seventh- to ninth-grade students	Having sex, unprotected sex, sexual initiation, number of sex partners
McBride et al., 2007 [90]	Quasi	U.S.A.	Community based	The Collaborative HIV/AIDS Adolescent Mental Health Project (CHAMP): the program involves having youth participate with parents and/or other adult caregivers who can steer them through pubertal changes, increases in romantic thoughts and feelings, and social pressure to engage in risky behavior, which may involve sexual activity.	Males and females aged 9–11 years	Parent–adolescent communication
Meekers, 2000 [91]	Quasi	South Africa	Community based	Targeted social marketing program on reproductive health beliefs and behavior	Young females	Knowledge and awareness
Meekers et al., 2005 [92]	Pre–post	Cameroon	Community based	“100% Jeune” social marketing campaign	Youth 15–24 years old	Perceived condom attributes and access, self-efficacy, and perceived social support
Merakou and Kourea-Kremastinou, 2006 [93]	Quasi	U.S.A.	School based	Peer education. Recruitment and training of the peer educators, implementation of HIV prevention activities in schools on behalf of the peer educators and evaluation.	13- to 17-year-old adolescents	Sexual encounter, condom use
Munodawafa et al., 1995 [95]	Quasi	Zimbabwe	School based	Student nurses to provide health instruction among rural school-age populations.	School-based adolescents	Knowledge
O-Donnell et al., 2005 [96]	RCT	U.S.A.	Community based	“Saving sex for later”—a parent and youth education program	Females and males aged 10–11 years	Communication
Ozcebe et al., 2004 [97]	Pre–post	Turkey	Community based	Peer education groups. An education program was scheduled every week and included the following discussion subjects: male and female anatomy–physiology of the reproductive system; types of STIs; etiopathology, progress and treatment of HIV/AIDS; preventive precautions against sexually transmitted diseases and HIV/AIDS; family planning methods; and communication skills.	Male and female adolescents	Knowledge
Pearlman et al., 2002 [98]	Quasi	U.S.A.	Community based	Peer education program. Short course and ongoing group work to plan HIV/AIDS outreach activities supervised by an adult.	Youth	Knowledge, perception, risk raking behaviors
Peltzer et al., 2011 [99]	RCT	U.S.A.	Clinic	Counseling, skills building: 18-minute information–education segment used a tabletop flip chart and visual materials to illustrate key concepts and interactive activities to dispute HIV myths, stigmas, and misinformation. The information component focused on HIV destigmatization as well as providing accurate risk information including risks related to male circumcision.	Males aged 18–35 years	Knowledge, number of sex partner, unprotected intercourse
Petersen et al., 2007 [100]	RCT	U.S.A.	PHC	Contraceptive counseling with optional advance EC prescription	Women aged 16–44 years	EC acceptance (only for 16- to 25-year age group)
Pinkleton et al., 2008 [101]	Quasi	U.S.A.	Community and school	Teen-led, media literacy curriculum focused on sexual portrayals in the media.	Primary- to middle-school students	Knowledge, efficacy, abstinence
Prado et al., 2012 [102]	RCT	U.S.A.	Community based	Family-specific interventions designed to reduce HIV risk behaviors	Hispanic males and females aged 12–17 years	Parent–adolescent communication, intercourse
Raymond et al., 2006 [103]	RCT	U.S.A.	Clinic based	Two methods of access to emergency contraceptive pills: increased access (two packages of pills dispensed in advance with unlimited resupply at no charge) or standard access (pills dispensed when needed at usual charges)	Females aged 14–24 years	Pregnancy, having sex, use of contraception, STI
Rickert et al., 2006 [104]	RCT	U.S.A.	Clinic	Contraceptive education and Depo Now compared to initiating with a bridge method (pills, transdermal patch, or vaginal ring)	Female aged 14–26 years	Consistent contraceptive use, hormonal contraceptive use, condom use, dual usage, no contraception, unintended pregnancy
Rocca et al., 2007 [105]	RCT	U.S.A.	Clinic based	Access to EC through advance provision, pharmacies, or clinics	Females aged 15–24 years	EC use
Rosenbaum, 2009 [106]	Quasi	U.S.A.	Community based	Virginity pledgers	>15 years of age virginity pledgers	Premarital sex, sexually transmitted diseases, and anal and oral sex variables
Ross et al., 2007 [107]	RCT	Tanzania	Community based	Community activities; teacher-led, peer-assisted sexual health education in Years 5–7 of primary school; training and supervision of health workers to provide “youth-friendly” sexual health services; and peer condom social marketing.	Adolescents	Knowledge, attitude, HIV, HSV
Rotheram-borus et al., 1998 [108]	RCT	U.S.A.	Community based	(1) Seven sessions of 1.5 hours each (10.5 hours); (2) three sessions of 3.5 hours each (10.5 hours); or (3) a no-intervention condition.	Adolescent aged 13–24 years	Self-efficacy, condom use.
Schreiber et al., 2010 [109]	RCT	U.S.A.	Clinic based	Routine postpartum contraceptive care and advanced supply of one pack of EC pills with unlimited supply thereafter upon request.	Postpartum teenage females	Sexual encounter, any contraception, condom use, EC use, pregnancy
Shrier et al., 2001 [110]	RCT	U.S.A.	Clinic based	Counseling and education. Standard STD education or to watch a videotape and have an individualized intervention session.	Female adolescents diagnosed with STI	Condom use, knowledge
Sieving et al., 2012 [111]	RCT	U.S.A.	Clinic	Counseling, skills building, and case management services.	Females aged 13–17 years	Number of sex partner
Suffoletto et al., 2013 [112]	RCT	U.S.A.	Community text messaging	Intervention participants received a sequence of text messages that assessed risky encounters over the past week, were provided personalized feedback on risk behavior, and were prompted collaborative goal setting to not have a risky encounter for the coming week.	Females aged 18–25 years	Condom use
Tocce et al., 2012 [113]	Quasi	U.S.A.	Clinic	Contraceptive education and etonogestrel implant (IPI)	Females aged 13–23 years	Consistent contraceptive use, hormonal contraceptive use, condom use, no contraception, unintended pregnancy
Villarruel et al., 2006 [115]	RCT	U.S.A.	School based	The HIV and health-promotion control interventions consisted of six 50-minute modules delivered by adult facilitators to small, mixed-gender groups in English or Spanish.	Latino adolescents aged 13–18 years	Self-reported sexual behavior
Villarruel et al., 2008 [114]	RCT	Mexico	Community	Parent education for adolescent sexual risk reduction	Parents of adolescents	Communication
Walker et al., 2004 [116]	RCT	Mexico	School based	An HIV prevention course that promoted condom use, the same course with emergency contraception as backup, or the existing sex education course.	High-school students	Knowledge, condom use
Weed et al., 2008 [117]	Quasi	U.S.A.	School based	Abstinence education program; the core of the program was a nine-unit abstinence curriculum taught consecutively over 20 class periods, called Reasonable Reasons to Wait: Keys to Character.	Seventh-grade adolescents	Sexual initiation
Wiggins et al., 2009 [118]	Quasi	England	Community based	Intensive, multicomponent youth development program including sex and drugs education (Young People's Development Program) versus standard youth provision.	13- to 15-year-old adolescents	Pregnancy, weekly cannabis use, and monthly drunkenness
Winter and Breckenmaker, 1990 [119]	Quasi	U.S.A.	Clinic	In-depth counseling and education in an adolescent friendly environment	Teenagers	Contraceptive use, pregnancy
Zimmer-Gembeck, 2001 [120]	Pre–post	U.S.A.	School based	Family planning care visits and contraceptive availability	Female adolescents	Use of contraception

AEC = advance emergency contraceptive; AIM = Adult Identity Mentoring; CImpact = Caribbean Informed Parents and Children Together; EC = emergency contraceptive; FSW = female sex workers; GFI = goal for information technology; HSV = herpes simplex virus; IPI = immediate postpartum transplant; OC = oral contraceptive; PHC = primary health care; RCT = randomized controlled trial; SMS = short messaging service; STD = sexually transmitted disease; STI = sexually transmitted infection.

**Table 2:** Summary of findings for the effect of sexual and reproductive health interventions

Quality assessment						Summary of findings		
Number of studies	Design	Limitations	Consistency	Directness		Number of participants		RR/SMD (95% CI)
				Generalizability to population of interest	Generalizability to intervention of interest	Intervention	Control	
Mean knowledge score: moderate outcome-specific quality of evidence								
13	RCT and pre–post	Study design is not robust	Twelve studies suggest benefit Substantial heterogeneity, I^2^ = 100%	All studies targeted adolescents	Multicomponent interventions	6,206	6,293	2.04 (1.31–2.78)
Mean efficacy score: moderate outcome-specific quality of evidence								
5	RCT and pre–post	Study design is not robust	Four studies suggest benefit Substantial heterogeneity, I^2^ = 99%	All studies targeted adolescents	Multicomponent intervention	2,699	2,508	.76 (.22–1.30)
Use of any contraception: moderate outcome-specific quality of evidence								
16	RCT and pre–post	Study design is not robust	Five studies suggest benefit Substantial heterogeneity, I^2^ = 89%	All studies targeted adolescents	Multicomponent intervention	9,269	9,364	1.07 (1.00–1.14)
Condom use: moderate outcome-specific quality of evidence								
23	RCT and pre–post	Study design is not robust	Seven studies suggest benefit Considerable heterogeneity, I^2^ = 72%	All studies targeted adolescents	Multicomponent intervention	9,659	9,842	1.11 (1.04–1.20)
Sexual encounter: moderate outcome-specific quality of evidence								
23	RCT and pre–post	Study design is not robust	Only two studies suggest reduced sex Substantial heterogeneity, I^2^ = 88%	All studies targeted adolescents	Multicomponent intervention	16,845	16,746	1.00 (.93–1.07)
Adolescent pregnancies: moderate outcome-specific quality of evidence								
18	RCT		Four studies showed significant improvement Some heterogeneity, I^2^ = 54%	All studies targeted adolescents	Comprehensive interventions addressing communities, sexual and reproductive health services, contraceptive provision and school-based education, and youth development	1,572	1,868	.85 (.74–.98)
Repeat adolescent pregnancies: moderate outcome-specific quality of evidence								
16	RCT		Six studies showed significant improvement Considerable heterogeneity, I^2^ = 74%	All studies targeted adolescents	Parental skills training and encouraging young mothers to finish school, as well as comprehensive medical care	1,572	1,868	.63 (.49–.82) [I^2^: 74%]
STI: moderate outcome-specific quality of evidence								
Six	RCT and pre–post	Study design is not robust	Only one study suggests reduced sex Considerable heterogeneity, I^2^ = 78%	All studies targeted adolescents	Multicomponent intervention	298	367	1.8 (.79–1.46) [I^2^: 78%]

CI = confidence interval; RCT = randomized controlled trial; RR = relative risk; SMD = standard mean difference; STI = sexually transmitted infection.

**Table 3:** Summary of findings for the effect of interventions to prevent female genital mutilation

Quality assessment						Summary of findings		
Number of studies	Design	Limitations	Consistency	Directness		Number of participants		SMD/RR (95% CI)
				Generalizability to population of interest	Generalizability to intervention of interest	Intervention	Control	
Belief that FGM/C compromise human rights of women: low outcome-specific quality of evidence								
One	Pre–post	Study design is not robust	Only one study	All studies targeted adolescents	Multicomponent intervention	1,120	1,120	1.30 (.47–3.64)
Prevalence of FGM/C: low outcome-specific quality of evidence								
Three	Pre–post	Study design is not robust	Two studies showed reduced prevalence. Low heterogeneity, I^2^ = 38%	All studies targeted adolescents	Multicomponent intervention	1,377	916	.63 (.49–.82)
Knowledge of harmful consequences of FGM/C: low outcome-specific quality of evidence								
Three	Pre–post	Study design is not robust	Two studies showed benefit Substantial heterogeneity, I^2^ = 98%	All studies targeted adolescents	Multicomponent intervention	2,368	1,987	1.53 (1.08–2.16)

CI = confidence interval; FGM/C = Female Genital Mutilation/Cutting; RR = relative risk; SMD = standard mean difference.
